# Integrating endothelium-derived hyperpolarization, nitric oxide, and prostacyclin pathways: the multimodal potential of human tissue kallikrein-1

**DOI:** 10.1530/VB-25-0006

**Published:** 2025-11-19

**Authors:** Paolo Madeddu, Styliani Goulopoulou, David Wambeke

**Affiliations:** ^1^ Experimental Cardiovascular Medicine, Bristol Medical School, Translational Health Sciences, University of Bristol, Bristol, United Kingdom; ^2^ Lawrence D Longo, MD Center for Perinatal Biology, Department Basic Sciences, Department Gynecology & Obstetrics, Loma Linda University, Loma Linda, California, USA; ^3^ DiaMedica Therapeutics, Minnetonka, Minnesota, USA

**Keywords:** endothelium, vasodilation, potassium channels

## Abstract

**Graphical Abstract:**

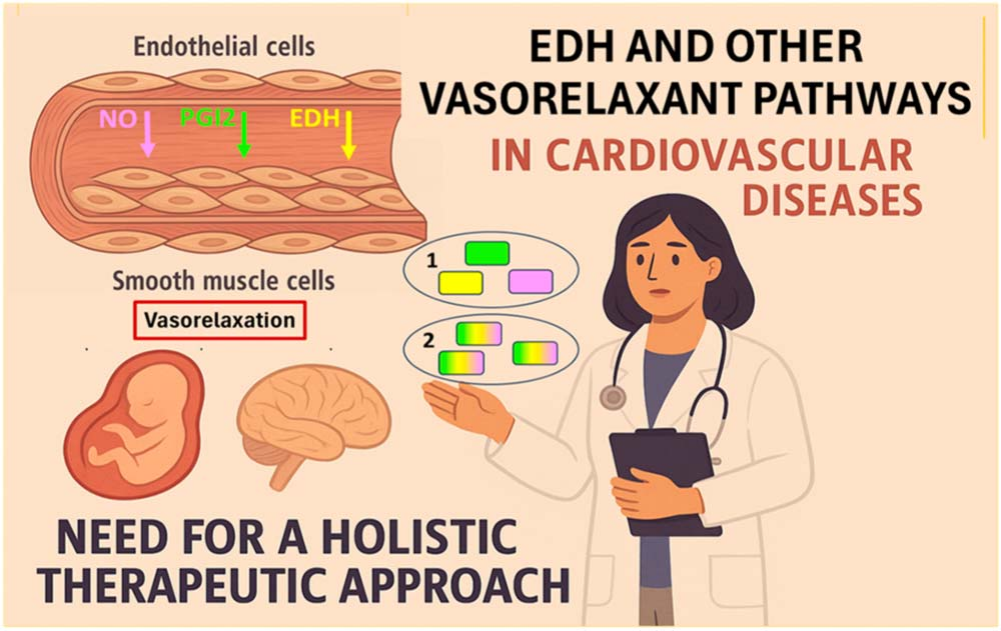

**Abstract:**

Endothelial cells regulate vascular tone by releasing nitric oxide (NO) and prostacyclin (PGI_2_), as well as by initiating hyperpolarization of vascular smooth muscle cells through K^+^ channels and myoendothelial coupling. This review highlights the therapeutic potential of targeting endothelium-dependent hyperpolarization (EDH) to address unmet needs in microvascular disorders such as cerebral small vessel disease, an important cause of stroke and dementia, and preeclampsia, a major pregnancy complication associated with maternal and fetal morbidity. Oxidative stress, connexin dysfunction, and impaired K^+^ channel signaling disrupt electrical coupling between endothelium and smooth muscle cells, leading to loss of vascular homeostasis. Building on this mechanistic convergence, we propose a multimodal therapeutic strategy to restore EDH in concert with the NO and PGI_2_ pathways. Within this framework, human tissue kallikrein-1 (KLK1) exemplifies an integrated therapeutic approach by simultaneously engaging multiple endothelial vasodilator mechanisms. Through bradykinin B_2_ receptor signaling, KLK1 enhances NO and PGI_2_ production while also promoting EDH via K^+^ channel activation. Its recombinant form, rinvecalinase alfa (DM199), has demonstrated consistent benefit in early-phase clinical trials, supporting its potential to restore endothelial balance. By reactivating these complementary vasodilatory pathways, DM199 improves microvascular perfusion and endothelial resilience, positioning it as a prototype multimodal therapy for microvascular diseases.

**Key message:**

Restoring endothelium-derived hyperpolarization alongside NO and prostacyclin signaling represents a promising multimodal approach to treat endothelial dysfunction and microvascular disorders.

## Introduction

For a long time, the vascular endothelium was considered an inert barrier separating the bloodstream from the vessel wall. This view changed dramatically with the discovery of endothelium-derived relaxing factors – nitric oxide (NO) and prostacyclin (PGI_2_) – which revealed the endothelium as an active, multifunctional organ essential to the regulation of vascular tone, homeostasis, and tissue perfusion (1, 2, 3, 4). Yet even when NO and PGI_2_ signaling are pharmacologically inhibited, blood vessels continue to relax in response to certain agonists (5, 6). This observation suggested the existence of additional endothelial mechanisms capable of sustaining vasodilation.

Among these mechanisms, endothelium-derived hyperpolarizing factor (EDHF) and endothelium-dependent hyperpolarization (EDH) have emerged as key contributors to vascular relaxation. EDHF refers to diffusible mediators – such as arachidonic acid metabolites, gaseous messengers, and reactive oxygen species – that induce hyperpolarization of vascular smooth muscle cells (VSMCs). EDH, by contrast, is mediated primarily through the activation of endothelial Ca^2+^-activated potassium (KCa) channels and electrical coupling with smooth muscle cells via myoendothelial gap junctions. These processes collectively promote vasodilation and enable resistance arteries to dynamically regulate local blood flow in response to metabolic demand.

The coexistence of multiple vasorelaxant systems – NO, PGI_2_, and EDH – confers an evolutionary advantage by providing redundancy and resilience against environmental or metabolic stress. The physiological relevance of this triad is finely tuned in an organotypic and angiotypic manner, allowing precise local control of blood flow across different organs and vascular beds. EDH plays a predominant role in the microcirculation, where it mediates conducted (ascending) vasodilation along arterioles to coordinate tissue perfusion (7, 8).

The first section of this review outlines the ion channels involved in EDH signaling. We then examine the connection between the cerebral and uteroplacental circulations, emphasizing that EDH is a key regulator of local blood flow and can be disrupted by oxidative stress, inflammatory mediators, and anti-angiogenic factors in conditions such as cerebral small vessel disease and preeclampsia. The following section addresses the translational gap in harnessing EDH therapeutically – potentially in concert with other vasorelaxant pathways. Preclinical studies suggest that activation of KCa channels can restore endothelial signaling and enhance microvascular reactivity, although safety concerns and off-target effects have limited clinical translation. Within this framework, we highlight human tissue kallikrein-1 (KLK1) and its recombinant form, DM199 (rinvecalinase alfa), as a promising example of a holistic therapeutic approach. Through bradykinin B_2_ receptor activation, KLK1/DM199 enhances NO and PGI_2_ production while simultaneously promoting EDH via KCa-channel activation. By restoring the coordinated activity of all three endothelial vasodilator pathways, DM199 has shown potential to improve microvascular perfusion and endothelial function in early clinical studies of ischemic stroke. Collectively, these findings position DM199 as a prototype multimodal therapy for microvascular dysfunction across diverse vascular territories.

## Conveyors of EDH-mediated vascular relaxation

### Potassium channels as central mediators

Electrical coupling between endothelial cells (ECs) and VSMCs depends on the opening of specialized potassium (K^+^) channels. These channels may be constitutively active or gated by membrane depolarization or elevations in intracellular Ca^2+^ concentration. Over recent decades, molecular characterization has expanded the known diversity of K^+^ channel families, prompting the International Union of Basic and Clinical Pharmacology (NC-IUPHAR) to propose a standardized nomenclature based on gene family and transmembrane domain number (6, 4, or 2) (9, 10, 11, 12).

### Ca^2+^-activated potassium channels (KCa)

The KCa channel family comprises three major subtypes – large-conductance (BKCa), intermediate-conductance (IKCa), and small-conductance (SKCa) channels – each with distinct physiological and pathological roles (11). BKCa (KCa1.1) and IKCa (KCa3.1) consist of single members, whereas the SKCa group includes three isoforms (KCa2.1, KCa2.2, and KCa2.3) (10, 11, 12, 13). Although KCa2.x and KCa3.1 share sequence similarities, they differ structurally from BKCa.

In most arterial beds, EDH-mediated vasorelaxation involves activation of endothelial SKCa (KCa2.3) and IKCa (KCa3.1) channels. BKCa channels, in contrast, are predominantly expressed in VSMCs and within the inner mitochondrial membrane of cardiomyocytes. These channels exhibit high K^+^ selectivity and single-channel conductance values approximately 10–20 times greater than those of other K^+^ channels. Their dual activation by depolarization and localized Ca^2+^ elevations allows rapid K^+^ efflux and membrane hyperpolarization that counteracts vasoconstriction (13, 14). BKCa channels thus provide a critical negative-feedback mechanism for VSMC contraction and contribute to physiological vasodilation. Altered expression or channel dysfunction – through either gain- or loss-of-function mechanisms – impairs vascular reactivity and has been implicated in hypertension, diabetic vasculopathy, and other endothelial disorders (15).

### Inward-rectifier potassium channels (Kir)

The inward-rectifier family includes constitutively active Kir2.x channels, G protein-activated Kir3.x channels, and ATP-sensitive Kir6.x channels that associate with sulfonylurea receptors. These channels form tetramers and may assemble as heteromers within subfamilies (e.g. Kir3.2 with Kir3.3). Kir channels are among the most prominent K^+^ channels in ECs and are also expressed by capillary pericytes.

Kir channels are activated by laminar shear stress, G protein-coupled receptor (GPCR) agonists, and K^+^ efflux from neighboring cells. The resulting hyperpolarization propagates through the endothelial layer to upstream feeding arteries, producing conducted vasodilation.

### Voltage-gated potassium channels (Kv)

Voltage-gated K^+^ (Kv) channels constitute an evolutionarily conserved family comprising twelve subfamilies. These channels are expressed in excitable tissues and within the vasculature, where they regulate resting membrane potential and vascular tone. Kv channels open at more negative potentials than KCa channels and are therefore particularly important in small resistance arterioles (16).

Within this group, Kv7 (KCNQ1–5) channels are especially relevant to vascular biology. Kv7 channels modulate cerebrovascular tone and preserve blood–brain barrier (BBB) integrity (17, 18). They also contribute to vasorelaxation in coronary and human chorionic plate arteries (19, 20).

### Hyperpolarization crosstalk between endothelial and smooth muscle cells

Activation of endothelial SKCa and IKCa channels induces membrane hyperpolarization and K^+^ efflux, which act as diffusible EDH signals. Electrical coupling through myoendothelial gap junctions (MEGJs), located within myoendothelial projections (MEPs), transmits this hyperpolarization to VSMCs. The process inhibits Ca^2+^ influx via voltage-gated Ca^2+^ channels (VGCCs), lowering intracellular Ca^2+^ levels, reducing myosin light-chain kinase (MLCK) activity, and thereby decreasing vascular resistance (21, 22).

K^+^ efflux and endothelial hyperpolarization also activate Na^+^/K^+^-ATPase and Kir channels, amplifying the hyperpolarizing response. Agonists such as acetylcholine and bradykinin trigger these pathways, producing electrical signals that propagate bidirectionally along the endothelium. The elongated morphology of ECs – spanning up to 20 smooth muscle cells – facilitates the longitudinal conduction of these hyperpolarizing currents.

### Integration with other vasodilatory mechanisms

Large conduit arteries (e.g. the aorta, carotid arteries, and major cerebral arteries) conduct blood with minimal resistance, whereas small arteries and arterioles (e.g. penetrating cerebral or uteroplacental spiral arteries) control local perfusion. NO-dependent signaling predominates in conduit arteries, whereas EDH-mediated vasodilation via KCa channels dominates in smaller resistance vessels.

NO and EDH exhibit a reciprocal relationship. Exogenous NO at physiological concentrations inhibits EDH-mediated relaxation in rabbit carotid and pig coronary arteries, likely by disrupting the synthesis or release of EDH-like factors (23). Conversely, inhibition or loss of endothelial NO synthase (eNOS) enhances EDH responses.

In small arteries, eNOS localizes to caveolae – specialized invaginations of the plasma membrane forming complexes with hemoglobin-α and cytochrome b_5_ reductase 3. Reducing hemoglobin-α increases NO scavenging, thereby limiting NO bioavailability. This microdomain is largely absent in conduit arteries, thereby increasing NO diffusion and bioavailability in those vessels (24, 25).

### Impact of risk factors on NO–EDH balance

Multiple cardiovascular risk factors impair NO bioavailability through convergent mechanisms. Intracellular L-arginine availability, regulated by argininosuccinate lyase, is rate-limiting for eNOS activity. In hypertension, diabetes, and hypercholesterolemia, eNOS becomes uncoupled, producing superoxide rather than NO and exacerbating oxidative stress. Uncoupling may arise from L-arginine or tetrahydrobiopterin (BH_4_) deficiency, excessive arginase activity, or accumulation of asymmetric dimethylarginine (ADMA) (26, 27, 28). Oxidative degradation of BH_4_, impaired eNOS dimerization, and glutathionylation similarly reduce endothelium-dependent vasodilation. Experimental eNOS uncoupling can be reproduced using competitive NOS inhibitors such as L-NAME or L-NMMA.

Depending on the disease context, EDH signaling may compensate for or contribute to endothelial dysfunction (29). This adaptability suggests that EDH serves as a flexible reserve vasodilatory mechanism when NO availability declines.

The following sections examine the pivotal role of EDH in two critical microvascular systems – the cerebral microcirculation and the uteroplacental circulation – which share structural and functional characteristics, including potent vasorelaxant capacity, endothelial plasticity, and specialized barrier function.

## A shared endothelial ‘language’ in two vascular compartments

Having presented the molecular conveyors of EDH, we next focus on two vascular systems in which this vasorelaxant mechanism serves as a master regulator of vascular tone. Both the cerebral and uteroplacental circulations are low-resistance, high-flow systems that depend on continuous endothelium-derived vasodilation, with limited NO diffusion and EDH mechanisms predominating. Hormonal modulation (estrogen, progesterone, relaxin) enhances EDH by upregulating KCa channels and connexins. When systemic endothelial dysfunction occurs (e.g. in preeclampsia), impaired EDH signaling reduces uteroplacental perfusion, resulting in placental ischemia and release of anti-angiogenic factors (e.g. sFlt-1), which disrupt cerebral endothelial function and EDH capacity, leading to cerebral vasospasm, impaired autoregulation, and risk of eclampsia. Hence, EDH is a mechanistic bridge; it ensures microvascular relaxation in both territories and fails in parallel when endothelial integrity is lost. Importantly, this framework reframes EDH not as a local vasodilator mechanism but as a systemic coordinating process – a paradigm shift in our understanding of global hemodynamic regulation. By integrating vascular tone across critical beds, EDH could represent a higher-order endothelial ‘language’ for distributing flow where it is most needed.

In the next sections, we present evidence that EDH regulates the two vascular districts separately and in combination, with potential implications for individualized therapeutic applications.

### The role of EDH in the regulation of small cerebral vessel relaxation

Cerebral arteries and arterioles continuously adjust their diameter to stabilize cerebral blood flow and protect the microcirculation from pressure fluctuations. The brain’s small vessels include cortical arteries that supply the cortex and deep-penetrating arteries that perfuse the basal ganglia and thalamus. Compared with large cerebral arteries, these smaller vessels exhibit a higher basal tone but retain a remarkable ability to dilate in response to neuronal activity. This neurovascular coupling ensures that local oxygen and nutrient delivery match metabolic demand.

Across the cerebrovascular network, NO-mediated vasorelaxation declines progressively with decreasing vessel size, whereas EDH-dependent relaxation increases, dominating within the microcirculation. Here, neurovascular and endothelial signaling converge through hyperpolarization to maintain perfusion in both physiological and pathological states. Capillary ECs detect neuronal activity and transmit electrical signals upstream to arterioles. Extracellular K^+^ released during neuronal firing activates Kir2.1 channels in capillary ECs, triggering rapid hyperpolarization that travels retrogradely to dilate upstream arterioles and pial arteries, thereby increasing capillary perfusion (30, 31).

### Alterations of K^+^ channels in cerebral small-vessel disease (SVD)

Cerebral SVD accounts for roughly 25% of all strokes and contributes to 45% of dementia cases. Its prevalence rises steeply with age – from ∼5% at age 50 to nearly 100% by age 90 (32). Characteristic vascular changes include wall thickening, disorganization, perivascular edema, and eventual microbleeds and lacunar infarcts. These structural alterations disrupt endothelial–smooth muscle communication and often precede BBB breakdown, compromising the brain’s protection against circulating toxins and pathogens (33).

Experimental evidence indicates that SKCa and IKCa channel activity reduces the basal tone of penetrating arteries but not larger cerebral vessels. During ischemia/reperfusion, levels of thromboxane A_2_ and peroxynitrite rise sharply, exerting opposite effects on EDH signaling: thromboxane activation suppresses hyperpolarization, whereas peroxynitrite enhances it. Despite eNOS inhibition during reperfusion, small arteries retain or even augment EDH-mediated dilation, suggesting a compensatory role for EDH when NO signaling is impaired (34).

Dysfunction of KCa channels has been linked to microvascular dementia. In APP23 mice, which overexpress amyloid precursor protein (similar to Alzheimer’s disease levels), pial arteries exhibit exaggerated myogenic constriction and reduced local Ca^2+^ release from ryanodine receptors, leading to decreased BKCa channel activity. The vasodilatory contribution of Kir2.1 channels is also diminished. Acute administration of amyloid-β_1–40_ to wild-type arteries reproduces the BKCa deficit without altering Kir2.1 function, implicating amyloid toxicity in BKCa impairment (35). Moreover, Kv7 channels, recently recognized as critical regulators of vascular tone, show reduced expression in hypertension, suggesting a broader role for voltage-gated K^+^ channels in cerebrovascular control (17).

EDH signaling may also influence BBB permeability through different ion channels. Both bovine and human BBB ECs express KCa3.1 currents and protein (36). During early ischemic stroke, cerebral edema results from Na^+^, Cl^−^, and water influx through the endothelium. Pharmacological inhibition of KCa3.1 channels within the first three hours of ischemia reduces this edema, indicating a therapeutic window (36). On the other hand, Kv7 channel activation in BBB ECs decreases permeability under normal and pathological conditions by promoting hyperpolarization and strengthening tight junctions, suggesting that Kv7 channel openers may protect against BBB disruption (18).

Together, these findings highlight EDH as a primary vasodilatory mechanism in the cerebral microcirculation. KCa and Kv7 channel activity help preserve perfusion during ischemia and hypertension, partially compensating for dysfunction of the NO pathway. However, excessive KCa activation in BBB endothelium may increase permeability and contribute to cerebral edema, whereas Kv7 activation appears protective.

### EDH signaling in the uteroplacental circulation during pregnancy

The uterine spiral arteries, terminal branches of the uterine artery, supply the placenta with oxygen and nutrients. During pregnancy, these vessels undergo extensive remodeling and functional adaptation to meet the demands of the growing fetoplacental unit (37, 38). As a result, uterine blood flow increases 10- to 20-fold – reaching 800–1,000 mL min^−1^ by term, or about 20% of cardiac output (39).

Remodeling follows an angiotypic program extending from large to small uterine arteries. In the decidua, endovascular trophoblasts replace the muscular and elastic layers of the spiral arteries with a fibrinoid matrix, lowering resistance and facilitating upstream vasodilation in radial and arcuate arteries (40). Functionally, uterine arteries become less responsive to vasoconstrictors and more sensitive to vasodilators, reflecting enhanced NO, PGI_2_, and EDH signaling (41).

In distal human myometrial arteries from both non-pregnant and late-pregnant women, EDH contributes substantially to endothelium-dependent dilation, as shown by residual relaxation to bradykinin and placental growth factor (PlGF) even when NO and prostaglandin synthesis are inhibited (42, 43, 44). Similar findings in rat radial uterine arteries confirm the increasing importance of EDH in controlling maternal vascular tone during pregnancy (45).

The relative contributions of NO, PGI_2_, and EDH vary across gestation. In late pregnancy, EDH-mediated vasodilation and associated reductions in smooth muscle Ca^2+^ are augmented, coinciding with enhanced endothelial Ca^2+^ levels and greater activation of SKCa and IKCa channels (46, 47, 48).

### Regulators of EDH in the uterine circulation

Several factors modulate EDH signaling during pregnancy. Estrogen is a major activator of SKCa channels via estrogen receptor α–mediated transcription of the KCNN3 gene encoding SKCa2.3. Upregulation of these channels increases Ca^2+^ sensitivity and enhances vasodilation. In ovariectomized rats, estrogen supplementation restores EDH responses (45), while prolonged 17β-estradiol treatment in sheep enhances eNOS, PKG-1α, and cGMP expression, leading to BKCa channel activation (49, 50, 51).

Vascular endothelial growth factor (VEGF) also promotes pregnancy-induced upregulation of SKCa2.3 and IKCa channels by preventing caveolin-mediated internalization. In cultured human uterine microvascular ECs, this VEGF-dependent increase is attenuated by VEGF-receptor blockade (52). Moreover, epigenetic modifications – including methylation of BKCa promoter regions – fine-tune KCa channel expression during normal and abnormal pregnancies (53).

### Altered K^+^ channel function in preeclampsia

Preeclampsia, affecting 5–8% of pregnancies worldwide, is a multisystem disorder defined by hypertension and proteinuria due to widespread endothelial dysfunction. It causes over 70,000 maternal and 500,000 infant deaths annually. Disease presentation varies from early (<34 weeks) to late-onset (>34 weeks) forms, each with distinct mechanisms (54). Early-onset preeclampsia often includes fetal growth restriction (FGR) due to inadequate placental perfusion (55, 56, 57). Maternal endothelial dysfunction extends beyond pregnancy, elevating long-term cardiovascular risk (58).

Reduced placental perfusion and syncytiotrophoblast stress trigger the release of pro-inflammatory, anti-angiogenic, and vasoactive mediators that impair endothelial barrier integrity and vasodilatory capacity (59). In this setting, KCa channel expression and activity decline, driven by oxidative stress, endoplasmic reticulum stress, and hypoxia-related epigenetic regulation (60). This loss of function disrupts hyperpolarization transfer through gap junctions in placental and umbilical vessels, compromising maternal and fetal vascular health (61).

Maternal undernutrition, a recognized preeclampsia risk factor, also diminishes EDH-mediated dilation in fetal coronary arteries. Under such conditions, bradykinin-induced relaxation becomes entirely NO-dependent, compensating for the loss of EDH (62).

Circulating anti-angiogenic factors further impair EDH. Maternal serum levels of soluble fms-like tyrosine kinase-1 (sFlt-1) rise, while VEGF and PlGF fall, promoting vasoconstriction and endothelial injury (63). Elevated sFlt-1 suppresses VEGF-driven SKCa and IKCa expression, while protein kinase C and ER stress further inhibit KCa activity (64). Collectively, these changes lead to maladaptive uteroplacental vascular remodeling and reduced perfusion.

### Brain–uteroplacental connection in preeclampsia

The cerebral and uteroplacental circulations use a similar ‘K^+^ channel language’, involving endothelial SKCa and IKCa in mediating the electrical coupling between ECs and VSMCs (60, 65). Moreover, recent human single-cell transcriptomic data have revealed overlapping expression of key endothelial and smooth muscle K^+^ channel genes in these two vascular systems, including KCNN3 (encoding SKCa2.3), KCNJ2 (Kir2.1), and KCNA5 (Kv1.5) (66). Table 1 illustrates the types and locations of shared channels.

**Table 1:** Potassium channels shared by cerebral and uteroplacental circulations.

Channel class	Primary localization	Cerebral role	Uteroplacental role
KCa2.3 (KCNN3)	Endothelium (microvessels)	EDH mediator; couples endothelial Ca^2+^ rises to smooth-muscle hyperpolarization via myoendothelial gap junctions; supports neurovascular coupling	Enhanced during normal pregnancy; facilitates low-resistance uterine artery tone; EDH compensation when NO signaling is limited (60)
KCa3.1 (KCNN4)	Endothelium (arterioles, resistance arteries)	Major EDH effector in cerebral microvessels; supports endothelium–smooth muscle electrical coupling	Upregulated by pregnancy hormones; critical to EDH in uterine arteries; blunted in preeclampsia models (65)
KCa1.1 (KCNMA1) + β/γ subunits	Vascular smooth muscle cells; placental endothelium	Controls smooth-muscle tone; contributes to dilation to shear/agonists; protects against pressure-induced constriction	Placental endothelial/smooth-muscle BKCa supports vasodilation; dysregulated in hypertensive pregnancy
Kir6.1/6.2 (KCNJ8/KCNJ11) + SUR2 (ABCC9)	Endothelium, smooth muscle cells, pericytes	Regulates cerebral blood flow during metabolic stress; contributes to collateral recruitment and flow reserve	Modulated by hypoxia/altitude; influences uterine artery vasodilation in pregnancy
Kv1.5 (KCNA5), Kv7 (KCNQ1/4/5)	Vascular smooth muscle cells; some endothelial expression	Sets the resting membrane potential and opposes vasoconstriction; participates in vasorelaxant responses to epoxyeicosatrienoic acids (EETs) following stimulation by bradykinin and acetylcholine or shear stress	Contributes to uterine artery tone regulation; pregnancy alters Kv7 signaling
Kir2.x (KCNJ2/4)	Endothelium, smooth muscle cells	Amplifies hyperpolarizing currents; involved in conducted vasodilation along parenchymal arterioles	Supports EDH spread and basal tone in uterine resistance vessels (evidence emerging) (66)
TREK-1 (KCNK2), TASK-1 (KCNK3)	Endothelium, smooth muscle cells	Mechanosensitive background K^+^ conductance; contributes to flow- and pressure-sensing in brain arterioles	Potential role in uterine artery mechano-sensing; limited but growing evidence

In preeclampsia, there is direct human evidence of EDH impairment and KCa channel downregulation in the uteroplacental circulation, accompanied by robust evidence of cerebral endothelial and BBB dysfunction and impaired autoregulation. Although direct measurements of cerebral K^+^ channel activity in patients with preeclampsia are not yet available, established EDH–K^+^ mechanisms governing neurovascular coupling and BBB control provide a coherent integrative framework. Loss of KCa-driven EDH likely contributes to placental hypoperfusion, while disrupted endothelial hyperpolarization and impaired BBB integrity promote cerebral edema and neurological complications.

This shared EDH blueprint across vascular beds supports the rationale for multimodal therapeutic strategies that restore SKCa/IKCa and Kv1.5/Kv7 channel function to protect both placental and cerebral microcirculations in preeclampsia. The nuclear factor erythroid 2–related factor 2 (Nrf2) has been shown to upregulate BKCa (KCNMA1) expression by binding directly to an antioxidant response element in the channel’s promoter region, suggesting that FDA-approved Nrf2 activators could enhance endothelial resilience in ischemic or hypertensive disorders (67). In addition, Kv7 channels may represent promising therapeutic targets to increase fetoplacental blood flow and protect the brain in preeclampsia (20).

Schematic illustration showing potassium (K^+^) channel subtypes expressed in endothelial and smooth muscle cells of both the cerebral and uteroplacental vasculature. Shared channels include voltage-gated (Kv), large-conductance calcium-activated (BKCa), inward-rectifier (Kir), ATP-sensitive (KATP), and two-pore domain (K_2_P) channels. These channels regulate membrane potential and vascular tone, promoting vasodilation and adaptive blood flow. Their overlapping expression highlights conserved mechanisms of microvascular regulation and suggests common therapeutic targets for hypertensive disorders of pregnancy. Strategies that preserve or enhance EDH signaling may offer dual protection for maternal and cerebral vascular health, positioning endothelial function as a key determinant of cardiovascular resilience.

## Emerging therapeutic approaches targeting K^+^ channels

### Positive gating modulators

Pharmacological strategies aimed at enhancing endothelial vasodilator pathways have mainly focused on supplementing eNOS substrates or cofactors, or, more commonly, using NO-donor drugs, soluble guanylyl cyclase (sGC) activators/stimulators (such as riociguat, cinaciguat, and vericiguat), and phosphodiesterase (PDE) inhibitors such as sildenafil. These compounds are particularly effective in conduit arteries, where endogenous NO signaling predominates (68, 69).

A growing area of interest lies in drugs that target endothelial ion signaling, particularly KCa channels, to restore microvascular blood flow. To date, however, no clinically approved drugs directly enhance hyperpolarization-mediated vasodilation. The first class of experimental compounds acts by activating KCa channels, exploiting their tissue-specific expression to achieve organ-selective effects (70, 71).

Positive gating modulators of KCa2.X/KCa3.1, such as NS309, stabilize the Ca^2+^-calmodulin-induced open state of these channels rather than directly activating them (72). This enhances the electrical response to a given cytosolic Ca^2+^ level. Early modulators (NS309, DCEBIO) were limited to *in vitro* use because of off-target effects and short plasma half-lives, but these studies demonstrated that enhanced hyperpolarization improves endothelial function (73, 74). Building on these findings, Wulff and colleagues developed a new class of KCa2.X/KCa3.1 modulators using riluzole as a scaffold, resulting in compounds with improved selectivity and bioavailability (75). The prototype, SKA-31, activates both IK1 and SK channels, producing vasorelaxation in phenylephrine-constricted resistance arteries (76), reducing blood pressure in normotensive and hypertensive mice, and improving endothelial and cardiac function in aged animals (77). Notably, low-dose SKA-31 (0.3 μM) enhanced acetylcholine- and bradykinin-induced vasodilation in resistance arteries from both diabetic rats and human subjects with type II diabetes, showing that endothelial KCa facilitation remains effective even after years of metabolic disease (78). Interestingly, SKA-31 activation of platelet KCa3.1 channels reduces platelet aggregation and adhesion, potentially stabilizing damaged or unstable endothelium (79).

### Complex roles of KCa channel modulation

In particular conditions, blockade rather than activation of KCa channels becomes desirable. Selective IKCa blockade prevents smooth muscle phenotypic switching and neointimal formation following angioplasty and during atherosclerosis (80). Inhibition of IKCa3.1 also reduces plaque formation and promotes anti-inflammatory monocyte polarization without systemic immune suppression. IKCa channels contribute to endothelial proliferation, tumor angiogenesis, and cancer cell migration; thus, their activation may carry pro-angiogenic risks (81). Unexpectedly, both KCa3.1 activators and inhibitors suppress smooth muscle proliferation, suggesting context-dependent effects possibly linked to differential expression or downstream signaling (82). SK3 (KCa2.3) channels are abundant in the central nervous system, but the outcomes of their chronic activation remain unclear. SKCa blockade has been proposed for treating cognitive disorders, depression, arrhythmias, and myotonic dystrophy (74).

### Additional molecular targets

Other promising ion-channel targets include Ca^2+^-release activated Ca^2+^ (CRAC) and transient receptor potential (TRP) channels. Although their contributions to vascular tone are incompletely understood, they represent underexplored therapeutic avenues. For instance, TRPV4 activation with the potent agonist GSK1016790A increases endothelial Ca^2+^ and induces vasorelaxation but paradoxically causes endothelial dysfunction and circulatory collapse (83).

In contrast, iptakalim, a selective ATP-sensitive K^+^ channel (KATP) opener targeting the SUR2B/Kir6.1 subtype, avoids these side effects. It preferentially dilates arterioles without affecting large arteries, reduces microvascular constriction, and mitigates no-reflow phenomena after ischemic stroke (84, 85).

## Integrated combination therapy targeting multiple vasorelaxant pathways

The previous section highlighted the approach of developing pharmaceuticals to activate or inhibit a specific pathway. The ‘one drug, one target’ model seeks to deliver potent and targeted therapeutic benefits while minimizing off-target side effects. However, this strategy may not be universally effective, as variations in key disease-related biological pathways across the general population could alter the pathogenic role of a particular target. In these instances, the influence of an alternative target may prevail, and such patients might derive more significant benefit from a combination therapy that concurrently targets both the primary and alternative pathways.

A multitarget approach using complementary pharmacological agents may better restore vascular function by engaging the NO, PGI_2_, and EDH pathways concurrently. This would address conditions resulting from combined macro- and microvascular disease. For example, significant vasodilatory mechanisms exhibit varying importance in cerebrovascular disease depending on vessel size, with vascular hyperpolarization dominating in smaller vessels and NO in larger ones. Large- and small-artery disease can coexist in the same patient, underscoring the need for combination therapies that target both districts. Among other benefits, drug associations result in fewer instances of drug resistance or evasion. As a result, medication combinations are now the norm for treating long-term illnesses such as cancer, inflammatory diseases, type II diabetes, bacterial and viral infections, and asthma.

Based on the connections between their targets, multitarget therapies can be divided into several groups. Therapeutic effects mediated by the drug combination’s impact on specific signaling pathways within the same or various cell types or tissues fall into the first category. To facilitate action at a second target level, the second category involves using a single medication to modulate a single target. Coordinated pharmacological activities at multiple sites on a single target or macromolecular complex fall under the third type. In many cases, the components of the combination are co-formulated into a single pill or injection; however, these combinations are often used as co-therapy regimens (86).

Typical examples are represented by multidrug treatment of resistant arterial hypertension (87), a polypill that includes aspirin, an angiotensin-converting–enzyme inhibitor, and a statin for the secondary prevention of cardiovascular death and complications after myocardial infarction (88), and NO-releasing statins combining 3-hydroxy-3-methylglutaryl-coenzyme A (HMG-CoA) reductase inhibition and slow NO release that possess more robust anti-inflammatory and antiproliferative activities than the native statins (89). Another example is Entresto, an oral combination that contains two blood pressure-lowering medications: sacubitril and valsartan. Sacubitril blocks neprilysin, an enzyme that degrades natriuretic (and other vasoactive) peptides, thereby relaxing blood vessels and promoting sodium and water excretion. Valsartan blocks the effects of the vasoconstrictor angiotensin II. Entresto is used to treat adults with heart failure to help reduce the risk of death and hospitalization, and to treat children aged 1 year and older who have symptomatic heart failure (90).

The Lacunar Intervention Trial-2 (LACI-2) randomized clinical trial was designed to deliver escalating doses of isosorbide mononitrate, a NO donor, and cilostazol, a phosphodiesterase enzyme (PDE3) inhibitor, which augments the prostacyclin-cAMP pathway in patients with small vessel stroke. The combination of these two drugs started months after the stroke and was given for over a year. Results indicate that the treatment reduced the risk of major vascular events and functional and cognitive outcomes compared to single-agent administration, with a good safety profile. These data support the concept that prolonged targeting of multiple vasorelaxant pathways via NO and prostacyclin may improve functional stroke recovery (91).

There are also examples of the therapeutic value of augmenting the NO–PGI_2_ signaling axis. Ohtuvayre® (ensifentrine) – a novel nebulized PDE3/4 inhibitor that enhances both cGMP and cAMP signaling – was approved by the FDA in 2024 for improving pulmonary function in adults with moderate to severe chronic obstructive pulmonary disease.

In summary, endothelial health depends on the synergy between NO, PGI_2_, and EDH pathways. Most vasodilator drugs target a single pathway – typically NO – but vascular protection requires balanced activation of all three pathways. Combining NO donors or sGC stimulators with selective KCa channel activators and agents supporting prostacyclin signaling could better preserve vascular homeostasis, particularly in hypertension, diabetes, and preeclampsia, where endothelial dysfunction is multifactorial.

## A multimodal, pathway-integrated approach may represent the next frontier in endothelial-targeted therapy

While drug combinations enhance the efficacy of disease treatment, they also present several disadvantages, including more side effects, excessive potency, limited dosage flexibility, poor patient adherence – particularly with separate formulations – and elevated costs. Therefore, it is preferable to utilize a single drug with multitarget capabilities.

Enzymes are natural catalysts that accelerate physiological reactions, such as the cleavage of vasoactive peptides from a substrate. The peptide’s interaction with its specific receptors activates various intracellular signaling pathways. This process facilitates intricate regulation of redundant pathways that have evolved to sustain physiological homeostasis, such as tissue perfusion. Herein, we present an example of a natural enzyme-peptide system that demonstrated therapeutic applicability in addressing tissue ischemia by activating multiple vasorelaxant pathways.

The serine protease tissue kallikrein (KLK1), extensively expressed in various tissues, including the vasculature, provides numerous protective effects against ischemia and promotes healing responses (92). KLK1 cleaves kinin peptides from the substrate low-molecular-weight kininogen (93). It is distinct from plasma kallikrein, another kinin-generating enzyme secreted by the liver as an inactive precursor (plasma prekallikrein). Both prekallikrein and factor XII (FXII) exhibit intrinsic proteolytic activity. They can activate each other in a reciprocal manner to generate FXIIa and plasma kallikrein, a process facilitated by a negatively charged surface during blood coagulation (94).

The expression and activity of KLK1 are reduced in patients with cardiovascular disease, and this deficit is associated with an increased risk of acute ischemic accidents such as stroke (95, 96, 97). Moreover, circulating maternal KLK1 levels are significantly lower in severe preeclampsia than in mild preeclampsia or normal pregnancy, and are negatively correlated with blood pressure and proteinuria (98). Reconstituting endogenous levels by exogenous administration of semipurified KLK1 prevented restenosis after stenting of severe atherosclerotic stenosis of the middle cerebral artery (99). In addition, urine-extracted KLK1 therapy has been successfully trialed in China on several hundred thousand stroke patients (100, 101). Nonetheless, the derivative nature of this medical product poses significant challenges for regulatory approval and patient acceptance outside of China.

Rinvecalinase alpha (DM199) is a recombinant version of the natural protein KLK1. DM199-based therapy addresses concerns about a semipurified protein. It has been titrated to maximize efficacy and safety. By restoring endogenous KLK1 levels and kinin production, DM199 activates multiple vasorelaxant pathways, all mediated by the kinin B2R. i) Under homeostatic conditions, kinin generation by KLK1 and B2R expression is set up to maintain physiologic NO release through IP3-dependent Ca2^+^ release from the endoplasmic reticulum. Following injury, such as brain ischemia, B2R expression is upregulated. Bradykinin stimulation generates prolonged high-output eNOS-derived NO (102). ii) Bradykinin activates the synthesis of PGI_2_ (103). An early report suggested that KLK1 can directly stimulate the release of PGI_2_ from vascular cells, independently of kinin formation, but through other substance(s) produced by this serine proteinase (104). iii) Bradykinin is also a potent activator of EDH signaling. The binding to the cognate B2R activates phospholipase C (PLC), an enzyme that hydrolyzes phosphoinositides. This hydrolysis releases inositol trisphosphate (IP3) and diacylglycerol (DAG). In turn, IP3 triggers the release of Ca2^+^ from intracellular stores, primarily the endoplasmic reticulum. The increase in intracellular Ca2^+^ concentration is a key step in activating KCa channels. This mechanism involves CaM binding to the C-terminal domain of KCa2.X/KCa3.1 channels, inducing pore opening and subsequent K^+^ efflux. In addition, in the coronary microcirculation, bradykinin can induce the release of a cytochrome P450-derived arachidonic acid metabolite that exhibits EDHF-like features (105). In the canine coronary artery, the hyperpolarization responses to bradykinin reportedly occurred at concentrations comparable to those initiating the NO-dependent component (106).

**Figure 1:**
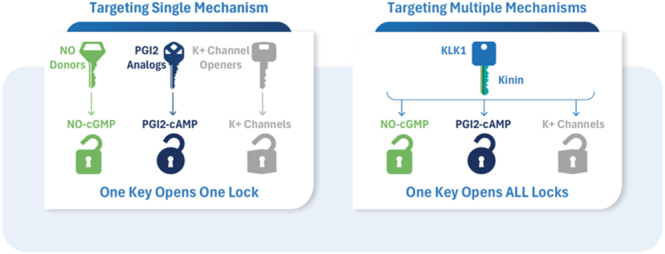
A master key to activate all vasodilatory mechanisms. KLK1-based therapy represents an advanced, multitarget vasodilatory approach in which the enzyme (the ‘bow’) generates kinins (the ‘master key’), which activate multiple vasoprotective mechanisms to restore vascular homeostasis. By enhancing endogenous kinin activity at the B2 receptor, it stimulates the coordinated release of NO, prostacyclin, and endothelium-derived hyperpolarizing factor – three synergistic pathways that collectively promote vasodilation, improve endothelial function, and enhance tissue perfusion. Preclinical studies in rodent models of cerebral, myocardial, and limb muscle ischemia provide strong mechanistic support for these effects. In isolated arteries, KLK1-induced dilation persists when either NO or PGI_2_ synthesis is inhibited but is abolished only when all three pathways are blocked. Through activation of endothelial calcium-activated potassium channels (KCa) – including small conductance (SKCa), intermediate conductance (IKCa), and large conductance (BKCa) isoforms – KLK1-induced bradykinin signaling enhances endothelial hyperpolarization and facilitates electrical coupling to vascular smooth muscle via connexin-mediated gap junctions (Cx37, Cx40, Cx43). These findings demonstrate that KLK1’s multitarget actions can simultaneously unlock several vasodilatory ‘locks’, offering a comprehensive and adaptive means of vascular protection that may translate into enhanced clinical efficacy. Reviewed in (92, 100, 101).

Pioneering research by Garland’s team demonstrated that bradykinin is a potent inducer of conducted (retrograde) hyperpolarization-dependent vasodilation, independent of NO or PGI2. When locally administered to human and porcine coronary arterioles, bradykinin evoked vasodilation that spread along the arteries with minimal decline for at least 1,000 μm from the application site. Endothelial SKCa and IKCa channels were responsible for this phenomenon (107). Interestingly, despite poor contractile function, diseased small coronary arteries showed preservation of conducted dilation in response to focally applied bradykinin. These data suggest that enhancing kinin generation by exogenous KLK1 administration can be therapeutically helpful in improving microvascular blood flow. Figure 1 illustrates using DM199-KLK1 as a master key to unlock the perfusion deficit.

## Early-phase clinical trials in stroke

The ReMEDy1 phase 2 clinical trial (NCT03795433) evaluated DM199 in patients with acute ischemic stroke who were ineligible for or beyond the therapeutic window for thrombolysis or thrombectomy (Table 2). Administered subcutaneously within 24 h of onset, DM199 demonstrated favorable safety and tolerability and produced encouraging signs of functional recovery. Interim analyses indicated that DM199 improved perfusion through collateral artery dilation within the ischemic penumbra, potentially extending the therapeutic window of stroke intervention. Moreover, patients treated with DM199 exhibited reduced risk of secondary strokes during follow-up, suggesting vascular stabilization beyond the acute phase.

**Table 2:** Summary of clinical studies of DM199 (recombinant human tissue kallikrein-1).

Study/model	Population/experimental system	Dose/route	Mechanistic focus	Key findings	Status/reference
DM199 (ReMEDy1 phase 2 trial, NCT03795433)	Acute ischemic stroke patients ineligible for tPA or thrombectomy; treatment within 24 h of onset	0.04–0.10 mg/kg SC every 12–24 h	Multimodal vasodilation via NO–PGI_2_–EDH	Safe and well-tolerated; improved collateral perfusion in ischemic penumbra; reduced risk of recurrent stroke; favorable trend in mRS at 90 days	Completed phase 2 (interim results 2023)
DM199 (ReMEDy2/planned phase 2b–3)	Acute ischemic stroke; broader inclusion including delayed-window and perfusion-mismatch patients	TBD (optimized from ReMEDy1)	Endotheliotropic vascular protection	Evaluating imaging-based perfusion outcomes, functional recovery, and biomarker response	In planning (FDA fast track 2024)
DM199 (investigator-sponsored trial in preeclampsia/FGR)	Pregnant women with early- or late-onset preeclampsia or fetal growth restriction	0.05–0.1 mg/kg SC daily (pilot dosing)	Restoration of SKCa/IKCa- and B_2_R-mediated EDH signaling in uteroplacental microcirculation	Ongoing: endpoints include maternal BP, uteroplacental Doppler flow, endothelial function, and fetal outcomes	Recruiting (planned completion 2026)

ACh, acetylcholine; BKCa, large-conductance Ca^2+^-activated K^+^ channel; B_2_R, bradykinin B_2_ receptor; EDH, endothelium-derived hyperpolarization; eNOS, endothelial nitric oxide synthase; FGR, fetal growth restriction; IKCa, intermediate-conductance Ca^2+^-activated K^+^ channel; KCa, Ca^2+^-activated potassium channel; KLK1, tissue kallikrein-1; mRS, modified Rankin Scale; NO, nitric oxide; PGI_2_, prostacyclin; SKCa, small-conductance Ca^2+^-activated K^+^ channel; SC, subcutaneous; tPA, tissue plasminogen activator.

A larger confirmatory phase 2/3 program is now under development to evaluate functional and imaging endpoints, including perfusion-weighted MRI, microvascular flow indices, and endothelial biomarker panels.

## Emerging clinical application: preeclampsia and fetal growth restriction

Building on these findings, an investigator-sponsored phase 2 study is currently evaluating DM199 in preeclampsia and FGR – two conditions characterized by endothelial dysfunction, placental hypoperfusion, and heightened vascular reactivity. The trial aims to determine whether DM199 can safely reduce maternal blood pressure, enhance uteroplacental perfusion, and improve fetal growth trajectories.

Mechanistically, this application is strongly supported by preclinical evidence that KLK1–bradykinin signaling activates KCa channels and restores EDH-mediated vasorelaxation in uteroplacental vessels. Restoration of SKCa/IKCa channel activity could improve trophoblast perfusion and maternal vascular compliance, while concurrent activation of NO and PGI_2_ pathways may further attenuate vascular constriction and inflammation. If successful, DM199 could represent the first disease-modifying therapy for preeclampsia – addressing both maternal hypertension and placental ischemia through a single, physiologically integrated mechanism.

## A translational bridge: from bench to bedside

Together, these studies illustrate how DM199 redefines endothelial pharmacology by simultaneously engaging three convergent vasodilator systems – NO, PGI_2_, and EDH – that operate across the cerebral, cardiac, and uteroplacental microcirculations. Its multimodal action offers a unified therapeutic approach to diseases previously treated through disparate single-pathway interventions.

Ongoing and future trials will clarify optimal dosing strategies, identify responsive patient subgroups, and determine whether biomarkers of endothelial function or KLK1 deficiency can guide precision use. If these studies confirm its benefits, DM199 may inaugurate a new class of ‘endotheliotropic’ multitarget therapeutics capable of restoring vascular homeostasis through a coordinated, systems-level mechanism.

## Conclusion

The proposal of a multitarget endothelial therapy (simultaneous modulation of NO, PGI_2_, and EDH) represents a systems-pharmacology approach to vascular health. This shifts the field from reductionist, single-pathway treatments toward network-based, compensatory therapeutics – a perspective that aligns with contemporary trends in vascular medicine and systems biology.

## Declaration of interest

PM is a scientific consultant for DiaMedica. PM is an Editorial board member on *Vascular Biology* but was not involved in the peer review process of this article. DW holds the Chief Business Officer position at DiaMedica.

## Funding

This work did not receive any specific grant from any funding agency in the public, commercial, or not-for-profit sector.
